# Editorial of the Special Issue: Recent Advances in Understanding of the Role of Synuclein Family Members in Health and Disease

**DOI:** 10.3390/biomedicines11092330

**Published:** 2023-08-22

**Authors:** Natalia Ninkina, Michail S. Kukharsky

**Affiliations:** 1 School of Biosciences, Cardiff University, Cardiff CF10 3AX, UK; 2 Institute of Physiologically Active Compounds at Federal Research Center of Problems of Chemical Physics and Medicinal Chemistry, Russian Academy of Sciences, 142432 Chernogolovka, Russia; kukharskym@gmail.com; 3 Department of Pharmacology and Clinical Pharmacology, Belgorod State National Research University, 308015 Belgorod, Russia; 4 Department of General and Cell Biology, Faculty of Medical Biology, Pirogov Russian National Research Medical University, 117997 Moscow, Russia

Extensive studies of α-synuclein function and dysfunction revealed its involvement in multiple normal and aberrant molecular processes and, consequently, numerous and diverse effects on the neuronal cell biology. The other two members of this family, namely β-synuclein and γ-synuclein, have also drawn increasing attention from researchers and clinicians over the past few years, but their roles in homeostasis and pathology of the nervous system are still poorly understood. Due to a high level of amino acid sequence homology, all three members of the synuclein family share many physicochemical properties. They also have overlapping patterns of intracellular localisation and expression throughout the nervous system. Taken together, these similarities point towards a possible functional redundancy within this family. However, each synuclein has its unique, i.e., unshared with the other two synucleins, functions, and in the context of certain cellular mechanisms and pathways, functions of these family members could be antagonistic rather than synergistic [1,2,3]. Therefore, it is important to contemplate the synuclein family as a conjoint protein system with a dynamic mode of functioning that requires fine tuning and balance to efficiently modulate the physiological processes these proteins are involved in. Moreover, dysfunction of the coordinated network of synucleins could trigger or critically contribute to the formation of pathomechanisms that lead to the development of certain neurological disorders. The number of research papers on synucleins that have been published since their discovery around 25 years ago has already exceeded 16,000. The articles published in the Special Issue “Recent Advances in Understanding of the Role of Synuclein Family Members in Health and Disease” add further value to this vast collection by addressing several aspects of synuclein biology and pathology, some of which are not in the mainstream of current research. The involvement of synucleins, particularly α-synuclein, in the pathogenesis of protein misfolding diseases (proteinopathies) has been the most thoroughly studied and well documented stream of data. The comprehensive review by A. Surguchov et al. [4] summarises the latest progress and main trends in research on the molecular mechanisms of synucleinopathies, a group of neurodegenerative diseases associated with the malfunction of synucleins. The current focus remains on the scrutinising processes of the pathogenic α-synuclein accumulation, aggregation, and formation of transmissible species that drive the aggregation pathology spreading across the nervous system. The authors of this review also covered the relatively recent studies that demonstrated the critical of role of epigenetics in the development of a synuclein-driven pathology. In vitro cellular models of synuclenopathies still remains a powerful tool for studying pathogenic fibrillation due to their throughput and fewer ethical issues faced. The capacity of this approach has been significantly reinforced through the advent of pluripotent stem cells and the ability to create mini brains in a dish. Katrina Albert and colleagues describe, in their review, how data that have been produced on various cellular models, such as immortalised cell lines, primary neurons, human-induced pluripotent stem cells (hiPSCs), blood–brain barrier models, and brain organoids, have substantially widened our knowledge of the mechanisms underlying synuclein aggregation and confirmed the crucial role of certain types of pathogenic fibrils in the triggering and transmission of proteinopathy from cell to cell, thereby allowing disease spread [5]. Several potentially important factors that could affect α-synuclein aggregation propensity have been recently identified. For example, α-synuclein fibrillation could be promoted through the receptor-binding domain of SARS-CoV-2 proteins [6]. Researchers have confirmed the formation of complexes between the receptor-binding domain (RBD) of the viral S-protein and the α-synuclein monomer following the immobilisation of RBD on its specific receptor ACE2. Several spectral approaches were used to produce novel data on the kinetics of amyloid fibril formation, which strongly suggested that the RBD prevents the amyloid transformation of α-synuclein. Moreover, the fibrils obtained in the presence of the RBD exhibited a significantly lower level of cytotoxicity on SH-SY5Y neuroblastoma cells. 

It is well known that synucleins are involved in the regulation of dopamine homeostasis [3,7,8,9,10]. The latest research revealed and delineated their role in the optimisation of dopamine uptake through synaptic vesicles and suggested that β-synuclein, rather than α-synuclein, potentiates this uptake [1,11]. These were discussed in connection with the different sensitivity of various synuclein deficient mouse models to MPTP toxicity [12]. In this review, the authors analysed available published data on how single, double, and triple knockouts of synuclein family members affect animal sensitivity to MPTP toxicity and attempted to explain the impact of α-, β-, and γ-synuclein on the mechanisms of MPTP and its active derivate, MPP+, toxicity on dopaminergic neurones. These authors also proposed a mechanistic explanation of why and how α-synuclein knockout mice demonstrated an increased resistance to MPTP, whereas the absence of β-synuclein attenuated this effect.

Consequences of the deletion of one or more synuclein genes and the resulting disbalance of these synucleins could manifest themselves in varied ways and modify certain physiological functions in model mice. For instance, Vorobyov et al. observed changes in electrical activity in the brains of synuclein knockout mice as their frequency spectra of electroencephalograms (EEGs) were being recorded [13]. EEGs were recorded from the motor cortex, the putamen, the ventral tegmental area, and the substantia nigra in mice lacking α-, β-, and γ-synucleins in all possible combinations, including completely synuclein-free triple knockout animals. Additionally, changes in the EEGs of these mice were assessed following the systemic injection of a DA receptor agonist, apomorphine (APO). The obtained data clearly demonstrated that it was not the absence of any particular synuclein, but rather that a disbalance of synucleins caused widespread changes in the EEG spectral profiles. Research by Kokhan et al. focused on studying the engagement of α-, β-, and γ-synucleins in mechanisms of craving for alcohol and developing alcohol dependence [14]. They described changes in the levels of expression in genes encoding for synucleins in the hippocampus and midbrain of alcohol-consuming male and female rats. Moreover, these authors revealed the sex-related differences in α-synuclein levels in the brains of adult rats that have been exposed to alcohol prenatally.

Due to the direct involvement of α-synuclein in the aetiology and pathogenesis of Parkinson’s disease, changes in the brain dopamine system are the main target for studies of pathological conditions caused by the malfunction of synucleins. However, the brain serotonin system is also affected by lesions in the synuclein network, and this could further exacerbate the patient’s health decline caused by problems with dopaminergic transmission. The serotonin pathways regulate mood and emotions, and therefore dysfunction of this system is crucial for the occurrence of certain non-motor symptoms in Parkinson’s disease patients at all stages of the disease progression, including in its very early stages, when a confident diagnosis is problematic. Consequently, better insight into how synuclein pathology affects the function of serotoninergic neurons is important for the development of novel therapeutic approaches for the treatment of non-motor symptoms that significantly affect patients’ quality of life. The most recent review by Miquel-Rio et al. summarised the latest progress and current knowledge about the involvement of α-synuclein in regulating serotonin system function in the context of health and disease [15]. The importance underlying the careful evaluation of non-motor symptoms, along with a multimodal MRI analysis, for assessing changes in brain function and managing patients with Parkinson’s disease and dementia with Lewy bodies, the two most common synucleinopathies, was also emphasised in the multimodal imaging study by Lucas-Jiménez et al. [16]. 

γ-synuclein is a well-known marker of certain types of malignant tumours; although, its association with neuronal pathology is less obvious than for the other two members of the family. However, increased levels of γ-synuclein expression in neurons causes its pathological aggregation and the development of severe neuronal pathology in transgenic mice [17,18,19]. Thus, it is feasible that a compensatory increase in γ-synuclein expression following the functional depletion of α- or/and β-synucleins could contribute to the nervous system malfunctioning. This may be linked to the appearance of autoantibodies against this protein in the CSF and peripheral blood of patients with certain disorders. For example, its expression and localisation are changed in the retina and optic nerves in patients with glaucoma. Moreover, Pavlenko et al. assessed the presence of autoantibodies to γ-synuclein in the blood serum of patients with primary open-angle glaucoma (POAG), and they have detected them in 20% of patients. Using γ-synuclein knockout mice as a model, they confirmed that γ-synuclein dysfunction contributes to pathological processes in glaucoma, including the dysregulation of intraocular pressure [20]. This is in accordance with the results of earlier studies that suggested the particular vulnerability of the visual system to malfunction of γ-synuclein due to the physiologically high levels of its expression in the retina and optic nerves [21].

Current therapeutic approaches to combat synucleinopathies are limited. Sai Sriram et al. have suggested, in their review, that α-synuclein aggregation pathology could be an actual target of deep brain stimulation (DBS), a surgical method that is currently in use for the treatment of three synucleinopathies: Parkinson’s disease (PD), dementia with Lewy bodies (DLB), and multiple system atrophy (MSA). In their review, these authors also discussed the usefulness and benefits of other surgical approaches, including focused ultrasound (FUS), for the management of these diseases [22].

The past few decades have been characterised by a sharp increase in the number of experimental and clinical studies of a group of severe and eventually fatal neurodegenerative diseases known as synucleinopathies due to the direct involvement of members of the synuclein family of proteins, primarily α-synuclein, in the aetiology and pathogenesis of these diseases. Although substantial progress has been achieved in understanding the molecular and cellular events associated with pathological changes in the nervous system typical to each of the synucleinopathies, our knowledge is still insufficient for the designing and creating of therapies that are capable of either preventing or halting the progression of these diseases. Therefore, the principal aim of current and future studies is to find out which components of these pathomechanisms are crucially responsible for the triggering and progression of these synucleinopathies, which would facilitate the development of efficient treatments for these diseases. Revealing an interplay of function and dysfunction of the synuclein family members in all its complexity and diversity would be an important future step in this direction.
